# Epidemiology, Distinct Seasonal Dynamics, and Age‐Specific Characteristics of Eight Respiratory Viruses in Thailand, 2025

**DOI:** 10.1155/jotm/8908857

**Published:** 2026-07-19

**Authors:** Nungruthai Suntronwong, Preeyaporn Vichaiwattana, Jiratchaya Puenpa, Siripat Pasittungkul, Ratchadawan Aeemjinda, Lakkhana Wongsrisang, Thanunrat Thongmee, Yong Poovorawan

**Affiliations:** ^1^ Center of Excellence in Clinical Virology, Department of Pediatrics, Faculty of Medicine, Chulalongkorn University, Bangkok 10330, Thailand, chula.ac.th; ^2^ The Royal Society of Thailand (FRS(T)), Sanam Sueapa Dusit, Bangkok 10330, Thailand

**Keywords:** acute respiratory infection, human rhinovirus, influenza, respiratory syncytial virus, respiratory virus, SARS-CoV-2

## Abstract

Acute respiratory infections (ARIs) impose a substantial annual public health burden, largely driven by a diverse range of respiratory pathogens. Given the dynamic and continuously evolving circulation of these pathogens, as well as their markedly different prevalence across age groups, ongoing surveillance is essential. To characterize the current prevalence, seasonal patterns, and age‐related distribution of eight key respiratory pathogens among ARI patients in Thailand, from January to December 2025, a retrospective study of 4697 respiratory samples from ARI patients aged 1 month to 97 years was conducted. Using real‐time RT‐PCR to screen for eight respiratory viruses, 61.1% (2871) of samples tested positive, with coinfections identified in 267 of the positive cases. Influenza virus (IFV) was the most prevalent pathogen at 22.2%, demonstrating a clear biannual pattern with peaks in January–March and July–December. Human rhinovirus (HRV) was the second most frequently detected virus (16.6%) and circulated year‐round, whereas SARS‐CoV‐2 accounted for 9.9% of cases, with a pronounced peak in May. Significant age‐specific differences in viral detection were observed. HRV predominated in children < 5 years, while IFV exhibited a broader distribution, with higher prevalence among individuals aged 6–18 years and ≥ 19 years. In contrast, SARS‐CoV‐2 was more frequently detected in adults and older populations. Coinfections were most common in children aged 3–5 years and were frequently associated with HRV. Notably, IFV, HRV, and RSV showed a significant increase in 2025 compared with 2024. These findings provide updated insights into the circulation of respiratory viruses in 2025, revealing distinct seasonal patterns, increasing influenza activity significantly with prolonged circulation in the latter half of the year, and clear age‐related differences in pathogen distribution. This provides effective support for continuous surveillance to inform effective public health strategies for ARI control.

## 1. Introduction

Acute respiratory infections (ARIs) impose a substantial annual burden on global public health, contributing significantly to hospitalizations and mortality across all age groups, particularly among young children and the elderly [1]. The severity of ARIs is influenced by multiple factors, including pathogen type, coinfection, patient age, and underlying comorbidities [2]. ARIs are etiologically diverse and involve a wide range of respiratory viruses, such as influenza virus (IFV), human adenovirus (HAdV), human rhinovirus (HRV), human parainfluenza virus (HPIV), respiratory syncytial virus (RSV), human coronavirus (HCoV), human metapneumovirus (HMPV), and, more recently, severe acute respiratory syndrome coronavirus 2 (SARS‐CoV‐2) [3].

The activity of respiratory viruses is shaped by a complex interplay of climatic conditions, population immunity, geographic factors, and human behavior, resulting in distinct seasonal patterns across regions and over time [4–6]. Unlike temperate regions, where infections typically peak during the winter months, respiratory virus activity in countries located within the intertropical belt, such as Thailand, often shows prolonged circulation, multiple peaks, or virus‐specific seasonal patterns [5, 7, 8]. The COVID‐19 pandemic has markedly disrupted global respiratory virus circulation, leading to delayed activity and shifts in the timing of seasonal onset [9, 10]. The subsequent resurgence of respiratory infections is likely driven by reduced immune stimulation due to decreased pathogen exposure and lower vaccine uptake, resulting in “immunity debt,” which increases population susceptibility following the lifting of pandemic restrictions [11–13]. In 2024, increasing trends in ARIs, including IFV, HRV, RSV, and HMPV, were observed in countries in the Northern Hemisphere, raising concerns about heightened respiratory activity in other regions [14]. Our previous study in Thailand in 2024 showed that respiratory viruses had begun to return to prepandemic seasonal patterns, cocirculating with SARS‐CoV‐2, with high activity observed during the warm and rainy months [15].

Although evidence is accumulating, continued surveillance is needed to determine whether respiratory viruses will return to their prepandemic seasonal patterns or establish new trends. This uncertainty represents an important gap in our understanding, particularly because accurately predicting the timing of outbreaks is essential for effective public health planning. For example, IFV vaccination programs are most effective when administered prior to peak transmission, while preventive interventions for high‐risk infants, such as those targeting RSV [16], need to be initiated at the onset of outbreaks. A clearer understanding of seasonal patterns and age‐specific viral distribution will improve healthcare planning, enhance protection for high‐risk populations, and reduce the overall burden of respiratory diseases.

This study investigated the epidemiology of eight respiratory viruses among patients with ARIs in Bangkok, Thailand. In a retrospective analysis covering January to December 2025, we used multiplex real‐time reverse transcription‐polymerase chain reaction (RT‐PCR) to analyze samples from patients of all ages. This work provides updated insights into the pathogen landscape, particularly in tropical settings where such patterns remain incompletely characterized. A clearer understanding of this epidemiology is essential for optimizing preventive strategies, improving control measures, and reducing the inappropriate antibiotic use that contributes to antimicrobial resistance.

## 2. Materials and Methods

### 2.1. Study Population and Ethical Approvals

This retrospective cohort study was conducted in Bangkok, Thailand, using data collected between January and December 2025. Eligible participants were individuals who sought care at Bangpakok 9 Hospital for ARIs, defined as fever (≥ 38°C) in combination with cough and/or sore throat. Nasopharyngeal swab specimens were collected and transported to the Center of Excellence in Clinical Virology, King Chulalongkorn Memorial Hospital, for routine respiratory virus surveillance testing. To avoid duplicate sampling, specimens collected from the same patient within a 14‐day interval or during follow‐up visits were excluded.

Ethical approval was obtained from the Institutional Review Board (IRB) of the Faculty of Medicine, Chulalongkorn University (IRB No. 0977/67), and the work adhered to the principles outlined in the Declaration of Helsinki and Good Clinical Practice standards. To maintain patient privacy, all personal identifiers were removed from the data before the analysis phase. The Institutional Review Board waived the need for individual informed consent due to the noninterventional, retrospective nature of the research. Permission to utilize the dataset was formally provided by the head of Bangpakok 9 International Hospital.

### 2.2. Viral Detection by Real‐Time RT‐PCR

Nucleic acids were extracted from 200 µL of clinical specimens using an automated extraction platform (magLEAD 12gC) (Precision System Science, Chiba, Japan), resulting in a final elution volume of 50 µL in accordance with the manufacturer’s protocol. Detection of respiratory viruses was performed using real‐time RT‐PCR assays as part of routine surveillance, as previously described [15]. Nucleic acid amplification assays were designed to target specific genes for each virus: the fusion (F) gene for HMPV, the hexon gene for HAdV, the N1 and N2 nucleocapsid genes for SARS‐CoV‐2, the matrix (M) gene for RSV and IFV, the hemagglutinin–neuraminidase gene for HPIV, the ORF1ab gene for HCoV, and the 5′ untranslated region for HRV.

To amplify RNA viruses, we used the SensiFAST Probe No‐ROX One‐Step Kit (Bioline, London, UK). Each 10‐µL reaction mixture contained 2 µL of extracted RNA (100 ng–1 µg), 5 µL of 2 × reaction mix, 0.4 µL of each primer (10 µM), 0.1 µL of probe, 0.2 µL of RNase inhibitor, 0.1 µL of reverse transcriptase, and nuclease‐free water. Thermal cycling began with reverse transcription at 42°C for 20 min, followed by initial polymerase activation at 95°C for 3 min and then 45 cycles of amplification, each consisting of 10 s at 95°C and 20 s at 60°C.

For the DNA virus (HAdV), the same reaction setup was used, except that RNA was replaced with 2 µL of DNA template and reverse transcriptase was omitted. The cycling conditions were identical, except that the initial reverse transcription step was excluded. Positive and negative controls were included in each run. In addition, the glyceraldehyde‐3‐phosphate dehydrogenase (GAPDH) gene was amplified as an internal control for sample quality. A sample was flagged as positive if its cycle threshold (Ct) value was 38 or lower.

### 2.3. Statistical Analysis

For our analysis, we grouped participants by age: infants (0–2 years), preschool‐aged children (3–5 years), school‐aged children (6–12 years), adolescents (13–18 years), adults (19–60 years), and older adults (> 60 years). The positivity rate for each epidemiological week (epi week) was defined as the percentage of positive specimens out of the total number tested. To analyze seasonality, we divided the year into three distinct climate periods: the warm season (March–May, Epi Weeks 10–22), the rainy season (June–October, epi week 23–44), and the cold season (January–February, epi week 1–9 and November–December, Epi Weeks 45–52). Because the number of samples tested per week was low in several age groups (< 10 samples/week), data were aggregated at the monthly level for the analysis of age‐specific positivity rates. The statistical analysis used either the chi‐square test or Fisher’s exact test to compare positivity rates across age groups. We also compared the viral detection rates observed in our study to the rates reported in 2024 using the chi‐square test. All data visualizations were created using R software (Version 4.5.1), while the statistical calculations were performed with IBM SPSS Statistics (Version 23.0). A *p* value of below 0.05 was considered statistically significant.

## 3. Results

### 3.1. Overall Prevalence of Respiratory Viruses and Coinfections

Our study analyzed 4697 nasopharyngeal samples collected throughout 2025 from patients who presented with acute respiratory symptoms. All samples were tested for all eight respiratory viruses, revealing a complex viral landscape. The study cohort comprised a slight majority of females (54.2%) and spanned a wide age range, from one month to 97 years. The mean age (SD) was 25.3 (23.3) years. In terms of age distribution, adults (19–60 years) represented the largest group at 37.9% (*n* = 1782), while adolescents (13–18 years) were the smallest at 6.4% (*n* = 302). Further details on the study population are provided in Table 1.

**TABLE 1:** Demographic data of participants and viral pathogen detection.

	Samples (%) (*N* = 4697)
Age, year (mean, SD)	25.3 (23.3)
Age group	
Infant (0−2 years)	540 (11.5%)
Preschool children (3−5 years)	632 (13.5%)
school‐aged children (6−12 years)	965 (20.6%)
Adolescents (13−18 years)	302 (6.4%)
Adults (19−60 years)	1782 (37.9%)
Older adults (> 60 years)	476 (10.1%)
Gender	
Female	2547 (54.2%)
Male	2150 (45.8%)

**FIGURE 1:**
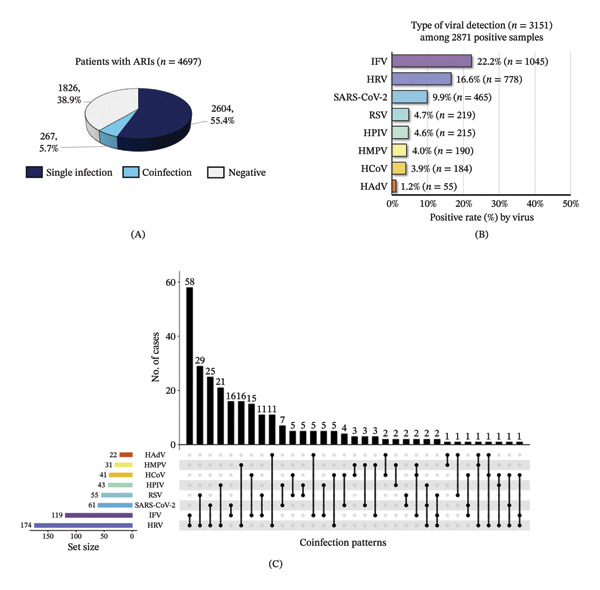
Prevalence and coinfection patterns of eight respiratory viruses among patients with acute respiratory infections (ARIs). A total of 4697 nasopharyngeal specimens collected from patients with ARIs between January and December 2025 were tested for eight respiratory viruses. (A) Proportion of ARI patients with single infections and coinfections. (B) Distribution of viral detections (*n* = 3151) among 2871 positive samples. The positivity rate for each virus was calculated as the number of samples testing positive for that virus divided by the total number of samples tested (*n* = 4697). The numbers beside each bar represent the corresponding positivity rates. (C) The UpSet plot illustrates the distribution of respiratory virus coinfection patterns. The top bars represent the number of cases for each unique coinfection pattern, while the left bars indicate the total number of detections for each virus. Connected dots denote the viruses comprising each coinfection pattern.

A total of 61.1% of individuals (2871/4697) tested positive for at least one of the eight respiratory viruses, underscoring the substantial burden of these pathogens (Figure 1A). Among all samples, single‐virus detections accounted for the majority of cases (55.4%, 2604/4697), while multivirus coinfections were identified in 5.7% of patients (267/4697). Among 2871 positive samples, 3151 viral detections were identified. IFV was the predominant pathogen, detected in 22.2% (1045/4697) of all samples (Figure 1B), followed by HRV and SARS‐CoV‐2, with detection rates of 16.6% (778/4697) and 9.9% (465/4697), respectively. Other notable respiratory viruses included RSV (4.7%), HPIV (4.6%), HMPV (4.0%), HCoV (3.9%), and HAdV (1.2%).

Analysis of coinfection patterns highlighted HRV as a key contributor to multiple‐pathogen infections. As shown in Figure 1C, the most common dual infections involved HRV in combination with other viruses, particularly, IFV (*n* = 58), RSV (*n* = 29), and SARS‐CoV‐2 (*n* = 21). Additionally, 12 cases of triple infections were identified, most frequently involving HRV and/or HCoV in combination with other viruses.

### 3.2. Seasonal Patterns for Eight Respiratory Viruses

**FIGURE 2:**
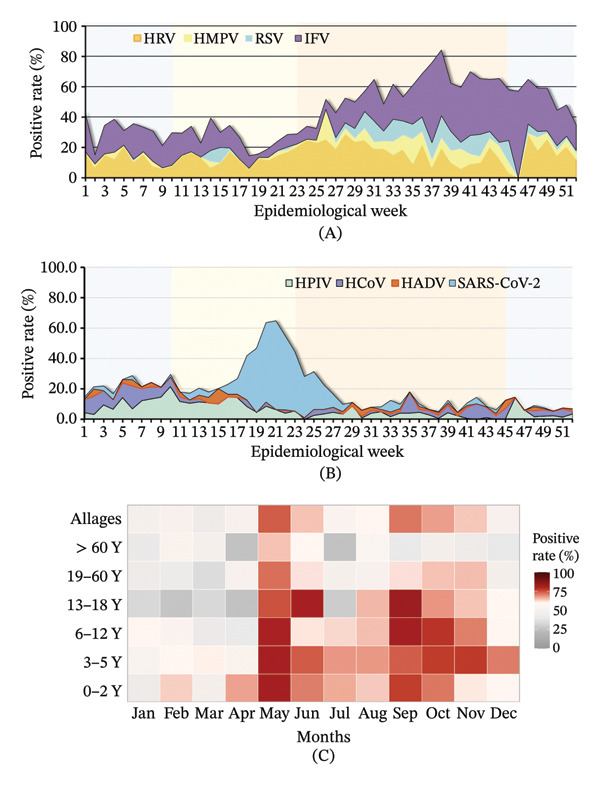
Weekly distribution of respiratory viral pathogens and monthly age‐specific positivity rates among patients with ARIs (*n* = 4697) in Bangkok, Thailand, from January to December 2025. Weekly positivity rates (*n* = 3151 positive samples) are shown for (A) human rhinovirus (HRV), human metapneumovirus (HMPV), respiratory syncytial virus (RSV), and influenza virus (IFV); and (B) human parainfluenza virus (HPIV), human coronavirus (HCoV), human adenovirus (HAdV), and SARS‐CoV‐2. Each virus is represented by a distinct color. Shaded areas indicate the seasonal periods: cool (January–February, Epi Weeks 1–9 and November–December, Epi Weeks 45–52), warm (March–May, Epi Weeks 10–22), and rainy (June–October, Epi Weeks 23–44). (C) Heatmap illustrating monthly viral positivity across different age groups. Abbreviation: epi week, epidemiological week.

To investigate seasonal patterns of respiratory viral infections, the weekly proportion of positive samples was analyzed for each pathogen. IFV activity exhibited two distinct peaks: a smaller increase during the cool season (January–March, Epi Weeks 1–13), with positivity rates ranging from 6% to 25.7%, and a more pronounced peak during the rainy season (July–December, Epi Weeks 27–52), reaching 13.5%–52.8%, with the highest peak in September (Epi Weeks 36–40) (Figure 2A). HRV circulated throughout the year, with positivity rates ranging from 3.5% to 29.5%. RSV and HMPV were primarily detected between July and September (Epi Weeks 27–40, rainy season), with concurrent peaks in August. In contrast, SARS‐CoV‐2 activity was highest during the warm months, peaking in May (Epi Weeks 19–22) (42.2%–48.6%) (Figure 2B). Other respiratory viruses, including HPIV, HCoV, and HAdV, were detected more frequently during the cooler months, although their overall prevalence remained relatively low.

Seasonal trends in viral detection across all age groups revealed two distinct peaks, occurring between May–June and September–November (Figure 2C). Children aged 0–2 and 3–5 years showed a more prolonged period of viral activity from May to November, with the highest detection rates observed in May and September. Among children aged 6–12 years, peaks were evident in May and September, whereas those aged 13–18 years showed peaks in June and September. In adults aged 19–60 years and older adults (> 60 years), viral detection was highest in May, although overall positivity rates in these groups were lower compared with younger age groups.

### 3.3. Age‐Specific Distribution for Eight Respiratory Virus and Coinfections

**FIGURE 3:**
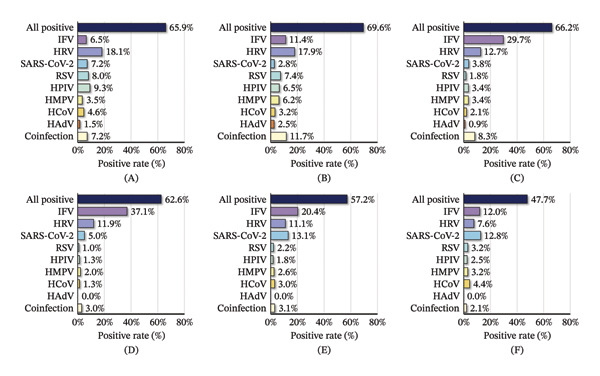
Distribution of viral positivity rates by age group. The proportions of eight respiratory viruses detected among (A) infants (0–2 Y) (*n* = 540), (B) preschool children (30–5 Y) (*n* = 632), (C) school‐aged children (60–12 Y) (*n* = 965), (D) adolescents (130–18 Y) (*n* = 302), (E) adults (190–60 Y) (*n* = 1782), and (F) older adults (> 60 Y) (*n* = 476) are shown. The numbers beside each bar represent the corresponding positivity rates. The positivity rate for each virus was calculated as the number of positive viral detections divided by the total number of samples in each age group.

We assessed differences in viral detection across age groups and found that the overall positivity rate was 65.9% (356/540) among infants (0–2 years), with HRV as the predominant virus, followed by HPIV and RSV (Figure 3A). In preschool children (3–5 years), 69.6% (440/632) of samples tested positive, with HRV, IFV, and RSV identified as the most common pathogens (Figure 3B). In school‐aged children (6–12 years), the positivity rate remained high at 66.2% (639/965), but the dominant virus shifted to IFV, with HRV and SARS‐CoV‐2 also being common (Figure 3C). Positivity rates for adolescents (13–18 years) and adults (19–60 years) were 62.6% (189/302) and 57.2% (1020/1782), respectively, and the viral landscape was consistently dominated by IFV, HRV, and SARS‐CoV‐2 (Figure 3D,E). Older adults (over 60 years) had the lowest rate of infection at 47.7% (227/476). In this age group, SARS‐CoV‐2 and IFV were the two most frequently detected viruses (Figure 3F). Overall, coinfections were most frequently observed in preschool children (11.7%), followed by school‐aged children (8.3%) and infants (7.2%).

### 3.4. Comparison of Viral Positivity Rates Among Age Groups

**FIGURE 4:**
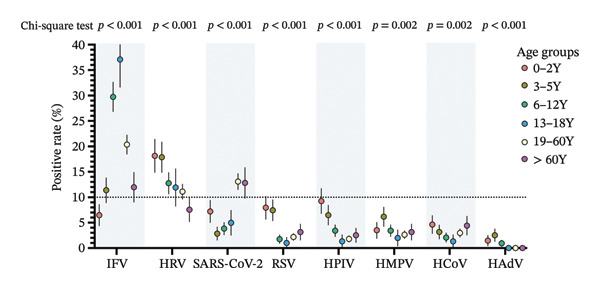
Comparison of viral positivity rates among age groups. The positive rates of eight respiratory viruses, including influenza virus (IFV), human rhinovirus (HRV), severe acute respiratory syndrome coronavirus 2 (SARS‐CoV‐2), respiratory syncytial virus (RSV), human parainfluenza virus (HPIV), human metapneumovirus (HMPV), human coronavirus (HCoV), and human adenovirus (HAdV), were compared across six age groups using the chi‐square test. A *p* value of < 0.05 was considered statistically significant.

Viral detection rates differed significantly across age groups. IFV was detected at a significantly higher rate among adolescents aged 13–18 years compared with other age groups (Figure 4). SARS‐CoV‐2 was most frequently detected in adults aged 19–60 years and older adults (> 60 years). In contrast, HRV, HPIV, RSV, and HCoV were detected at significantly higher rates in infants aged 0–2 years. HAdV and HMPV were most commonly detected among preschool children aged 3–5 years.

### 3.5. Temporal Shifts in the Viral Landscape Between 2025 and 2024

**FIGURE 5:**
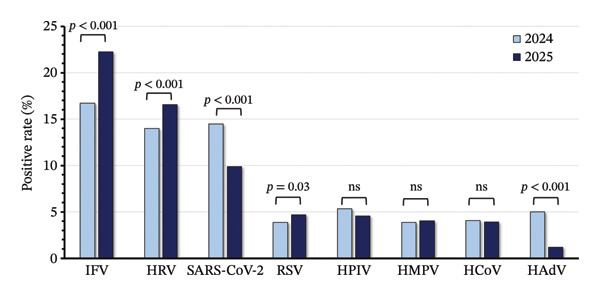
Comparison of viral positivity rates between 2024 and 2025. The positivity rate of each virus in 2025 was compared with that in 2024. Statistical significance was assessed using the chi‐square test, with *p* < 0.05 considered statistically significant. ns indicates not statistically significant.

To assess postpandemic changes in respiratory virus epidemiology, we compared viral positivity rates in 2025 with those from our 2024 cohort [15]. This analysis revealed a marked shift in the viral landscape (Figure 5). A notable resurgence was observed, with significantly higher positivity rates of IFV, HRV, and RSV in 2025. In contrast, SARS‐CoV‐2 and HAdV declined significantly. Meanwhile, no significant differences were observed in the detection rates of HPIV, HMPV, and HCoV, indicating stable circulation between the two years.

## 4. Discussion

This study evaluated the epidemiology of eight respiratory viruses among patients with ARIs in Thailand over a one‐year period (January–December 2025). Overall, viral pathogens were detected in 61.1% of cases, highlighting the substantial contribution of respiratory viruses to ARIs across all age groups. Among the viruses tested, IFV, HRV, and SARS‐CoV‐2 were the most frequently identified, consistent with global trends in respiratory virus infections [17, 18]. Increased IFV activity in the latter half of the year was consistent with patterns observed in other countries in Southeast Asia [19] and was higher and more prolonged than that reported in our previous 2024 study [15]. Notably, detection rates were higher in pediatric populations than in adults and older individuals, with distinct viral profiles observed across age groups. Coinfections were also more common in children, frequently involving HRV in combination with other pathogens, consistent with previous studies [15, 20].

Overall, clear seasonal patterns in viral circulation were observed throughout the year, with peaks in May and September. SARS‐CoV‐2 activity increased earlier in the year, followed by rises in IFV, RSV, and HMPV, while HRV circulated year‐round. In contrast, other respiratory viruses showed relatively low and less consistent activity. These temporal patterns were largely consistent with those reported in 2024, with minor shifts in peak timing. SARS‐CoV‐2 activity was delayed from April to May, while IFV activity shifted its highest peak from July to September and showed a more prolonged period of circulation compared with that reported in 2024 [15]. Such shifts may be associated with behavioral factors, including increased social gatherings during national events, as well as the emergence of new viral variants [21, 22]. Comparisons with global analyses of postpandemic peak timing suggest that the patterns observed in the present study differ from those reported in other countries within the intertropical belt [9]. Although Thailand is located in this region and shares a similar climate with neighboring countries such as Laos PDR, Cambodia, Myanmar, Malaysia, and Vietnam, respiratory virus seasonality varies across these settings [5]. For example, IFV activity in Cambodia, Laos PDR, and Myanmar typically exhibits a single distinct peak, whereas Thailand often shows a bimodal pattern [23]. In contrast, Malaysia and Vietnam demonstrate more continuous, year‐round activity [24]. These variations underscore the importance of continued surveillance, as country‐specific seasonal patterns are shaped by a complex interplay of local climate, population immunity, geographic factors, and social behavior.

Age‐specific analyses further highlighted the differences in virus distribution across age groups. Children exhibited the highest overall detection rates, consistent with their increased susceptibility to upper respiratory tract infections [25]. In particular, HRV was the predominant pathogen among infants (0–2 years) and preschool‐aged children (3–5 years), whereas it was the second most frequently detected pathogen in all other age groups, consistent with reports from the United States [26], the United Kingdom [27] and China [28]. Young children are also key drivers of rhinovirus transmission within households [29]. Although most HRV infections are mild, they are commonly associated with acute bronchiolitis and asthma exacerbations [30]. Consistent with previous reports, RSV, HPIV, and IFV were frequently detected among children aged < 5 years and are associated with an increased risk of severe disease [31, 32]. Evidence from South Africa further highlights the clinical impact of RSV, where it accounted for 31% of lower respiratory tract infection (LRTI) hospitalizations overall, and 22% of all‐cause hospitalizations and 41% of LRTI hospitalizations among infants younger than six months [33]. The high prevalence of RSV in children is supported by established immunological mechanisms, including the waning of maternally derived antibodies during the first year of life, subsequent seroconversion following primary infection [34], and a high reinfection rate (34.4 per 1000 children aged ≤ 4 years) [35].

School‐aged children (6–12 years) and adolescents (13–18 years) were more frequently affected by IFV and HRV. This predominance likely reflects increased transmission following school reopening, driven by close contact among students, prolonged classroom exposure, and poor indoor air quality [36]. In contrast, adults and older adults were more frequently infectedwith IFV and SARS‐CoV‐2, consistent with a previous study indicating a higher burden of these viruses in adult populations [37]. The IFV positivity rate in 2025 was higher than that reported in our 2024 study [15]. Similarly, increased IFV activity has been observed in England [38], the United States [39], and Australia and New Zealand [40]. This rise may be associated with the emergence of new subclades with amino acid substitutions that potentially enable immune escape from vaccine‐induced protection [22]. These findings further underscore the need to strengthen ongoing molecular and epidemiological surveillance.

The substantial detection of HRV, IFV, RSV, and HPIV in pediatric populations underscores the critical importance of preventive strategies, including vaccination and prophylactic interventions, to reduce pressure on healthcare systems, particularly in low‐ and middle‐income settings where disease severity is often greater. Currently, IFV vaccines are available for children aged ≥ 6 months and have been shown to reduce the risk of infection among school‐age children [41]. Although active immunization against RSV is not yet available for young children, passive immunization options have been approved for prophylaxis, including long‐acting monoclonal antibodies such as nirsevimab, palivizumab, and clesrovimab [42]. In addition, vaccines targeting the prefusion RSV protein have been licensed for maternal immunization to protect infants from RSV‐associated LRTI [16]. While no licensed vaccines are currently available for HRV, HPIV, or HMPV, several candidates are under development [43–45]. Among adults and older individuals, the predominance of IFV and SARS‐CoV‐2, particularly in those at higher risk of severe disease, highlights the importance of vaccination as a key public health strategy. Vaccines for both IFV and SARS‐CoV‐2 are widely available, and although their effectiveness may be influenced by emerging variants, they remain highly effective in reducing disease severity [46, 47]. Overall, characterizing age‐specific detection patterns and seasonal trends is crucial for optimizing the timing and implementation of preventive measures, such as vaccination campaigns and RSV monoclonal antibody prophylaxis, thereby enhancing their effectiveness in reducing the burden of respiratory diseases.

The novelty of the present study lies in its extended surveillance through 2025, enabling the capture of newly stabilized patterns of respiratory virus circulation in the postpandemic era. This extended timeframe allows for a more definitive characterization of seasonal trends that were previously uncertain [15]. However, several limitations should be acknowledged. First, as a single‐center study conducted at a tertiary‐care hospital in Bangkok, the findings may have limited generalizability to other regions of Thailand. Second, the one‐year study period limits the ability to fully assess long‐term seasonal variability, particularly, for SARS‐CoV‐2 in the postpandemic context. Continued multiyear surveillance is needed to determine whether the observed patterns reflect stable trends or short‐term fluctuations. Third, this study focused exclusively on viral pathogens and did not assess bacterial causes of ARIs, which, although less frequently detected, may still contribute to the overall disease burden [48]. Additionally, IFV‐positive specimens were not further subtyped into A/H1N1pdm09, A/H3N2, or influenza B lineages, preventing more detailed assessment of strain‐specific epidemiology. Finally, the retrospective laboratory‐based design and lack of detailed clinical outcome data restricted the evaluation of disease severity and risk factors associated with infection.

In conclusion, this study provides a comprehensive characterization of respiratory virus circulation in Bangkok, Thailand, in 2025, revealing distinct seasonal patterns, heightened IFV activity with prolonged circulation in the latter half of the year, and clear age‐related differences in pathogen distribution. These findings offer important public health insights and support the development of targeted prevention strategies, more effective clinical management during peak transmission periods, and optimized vaccination policies to reduce the burden of respiratory infections.

## Author Contributions

Conceptualization, Nungruthai Suntronwong and Yong Poovorawan; data collection, Preeyaporn Vichaiwattana, Jiratchaya Puenpa, Siripat Pasittungkul, Ratchadawan Aeemjinda, Lakkhana Wongsrisang, and Thanunrat Thongmee; formal analysis, Nungruthai Suntronwong; methodology, Preeyaporn Vichaiwattana, Jiratchaya Puenpa, Siripat Pasittungkul, and Ratchadawan Aeemjinda; project administration, Yong Poovorawan; writing–original draft, Nungruthai Suntronwong; and writing–review and editing, Nungruthai Suntronwong and Yong Poovorawan.

## Funding

This work was supported by Health Systems Research Institute, the Center of Excellence in Clinical Virology of Chulalongkorn University, and King Chulalongkorn Memorial Hospital, the MK Restaurant Group and Aunt Thongkham Foundation. Nungruthai Suntronwong reports that financial support was also provided by the Second Century Fund Fellowship of Chulalongkorn University.

## Disclosure

All authors have read and agreed to the published version of the manuscript.

## Conflicts of Interest

The authors declare no conflicts of interest.

## Data Availability

The data that support the findings of this study are available from the corresponding author upon reasonable request.
